# Predictors of Antihypertensive Drug Adherence and Blood Pressure Control Among Hypertensive Patients: A Multicenter Cross-Sectional Study

**DOI:** 10.1155/ijhy/1055517

**Published:** 2025-08-28

**Authors:** Tamrat Petros Elias, Asteraye Tsige Minyilshewa, Mengesha Akale Tekle, Tsegaye Wesenseged Gebreamlak, Binyam Lukas Adde

**Affiliations:** ^1^Department of Internal Medicine, St. Paul's Hospital Millennium Medical College, Addis Ababa, Ethiopia; ^2^Department of Internal Medicine, Yekatit 12 Hospital Medical College, Addis Ababa, Ethiopia; ^3^Department of Internal Medicine, Adera Medical and Surgical Center, Addis Ababa, Ethiopia; ^4^Department of Internal Medicine, Menelik II Comprehensive Specialized Hospital, Addis Ababa, Ethiopia

**Keywords:** adherence, antihypertensive drugs, blood pressure control, hypertension

## Abstract

**Background:** Hypertension or elevated blood pressure is a serious medical condition that significantly increases the risk of diseases of the heart, brain, kidneys, and other organs. Antihypertensive drug adherence is key to controlling blood pressure. This study aimed to assess factors associated with antihypertensive drug adherence and blood pressure control among hypertensive patients in selected public hospitals under the Addis Ababa City Administration.

**Method:** A hospital-based cross-sectional study was conducted among hypertensive patients on follow-up in randomly selected public hospitals under the Addis Ababa City Administration from November 1, 2022, to February 28, 2023. The study population included 393 patients who fulfilled the eligibility criteria and were selected by systematic random sampling. Data collection was conducted from the electronic medical records and by interviewing patients with a structured questionnaire. The data were entered into Epi-Info 7.2.1 and exported to SPSS version 25 software for analysis. Logistic regression analysis was performed to determine the associations between the dependent and independent variables.

**Results:** The rates of antihypertensive drug adherence and blood pressure control were 72.5% and 23.4%, respectively. Participants with uncontrolled blood pressure were 41.7% less adherent than those with controlled blood pressure (AOR = 0.59; 95% CI, 0.36–0.97). Nonadherence to dietary restriction (AOR, 3.31; 95% CI, 1.84–5.96) and chronic kidney disease (AOR = 3.85; 95% CI, 1.41–10.52) were associated with good adherence, whereas the use of a single antihypertensive drug (AOR = 0.53; 95% CI, 0.30–0.94) and nonadherence to moderate physical exercise (AOR = 0.30; 95% CI, 0.20–0.65) were associated with poor adherence to antihypertensive medications. Male sex (AOR = 1.95; 95% CI, 1.04–3.28) and blood pressure measured at home (AOR = 0.59; 95% CI, 0.36–0.99) were found to be independent predictors of controlled blood pressure. Drinking alcohol (AOR = 1.92; 95% CI, 1.05–3.49) was inversely associated with blood pressure control.

**Conclusion:** Although adherence to antihypertensive medications was relatively good, blood pressure control remained low, indicating that medication adherence alone is insufficient. Public health policies should focus on strengthening primary care systems to deliver integrated hypertension management, including lifestyle counseling, dietary support, and improved access to medications and monitoring tools.

## 1. Introduction

Hypertension or elevated blood pressure is a serious medical condition that can be defined as office systolic blood pressure (SBP) ≥ 140 mmHg and/or diastolic blood pressure (DBP) ≥ 90 mmHg, or a BP at which the benefits of treatment unequivocally outweigh the risks of treatment [1].

In 2019, an estimated 1.3 billion people worldwide had high blood pressure, and it is estimated that the number of people with hypertension will increase by 15%–20% by 2025, reaching close to 1.5 billion [2, 3]. The overall prevalence of hypertension in adults is approximately 30%–45% [4]. The estimated pooled prevalence of hypertension is approximately 30.8% in Africa [5] and 30.0%–31.1% in Sub-Saharan Africa [6]. In Ethiopia, there are different prevalence reports in different studies and across different regions of the country. Systematic meta-analyses conducted in 2019 using published and unpublished articles showed that the overall pooled prevalence of hypertension in the country was 21.8% [7].

Hypertension is associated with a substantial financial burden. The global financial burden of high BP in 2001 was estimated to be approximately US$370 billion, or approximately 10% of the world's overall healthcare expenditure [8].

Elevated BP is also associated with a large global burden of cardiovascular disease (CVD) and premature death. According to data from different studies, an estimated 7.7–10.4 million annual deaths are attributable to elevated blood pressure levels, 88% of which are in low- and middle-income countries (LMICs) [9–11].

Randomized clinical trials have demonstrated that lowering BP with commonly used regimens, such as diuretics, angiotensin-converting enzyme (ACE) inhibitors, angiotensin receptor blockers (ARBs), and calcium channel blockers (CCBs), reduces the risk of CVD and all-cause mortality [12, 13].

Despite these effective interventions, hypertension control remains unacceptably low, particularly in LMICs. Recent global estimates suggest that only 45.6% of people with hypertension are aware of their condition; only 36.9% are receiving treatment, and only 13.8% achieve BP control (defined as SBP < 140 mmHg and DBP < 90 mmHg) [14].

In chronic diseases, including hypertension, medication mainly serves as a preventive measure and does not suppress symptoms; maintaining long-term medication adherence is particularly difficult, and the risk of treatment discontinuation is very high [15].

Various studies have shown that poor medication adherence is a primary reason for poor BP control [16, 17]. Poor adherence is responsible for unnecessary over prescription of drugs, substantial worsening of disease, increased hospital admission rates, and longer hospital stays, all of which lead to a significant medical burden, such as reduced optimal clinical benefit and increased risk of cardiovascular events, including acute coronary syndrome, stroke, chronic heart failure, and mortality [18]. In addition to adverse cardiovascular events, it is also associated with reduced quality of life, disability, reduced work productivity, and greater healthcare costs [19].

The purpose of this study was to assess the adherence rate and blood pressure control among hypertensive patients in selected public hospitals under the Addis Ababa city administration and to identify factors associated with poor adherence and blood pressure control. Therefore, knowing the magnitude and reasons for the problem, appropriate interventions can be planned to improve adherence and blood pressure control.

## 2. Materials and Methods

### 2.1. Study Design and Setting

A hospital-based cross-sectional study was conducted at Yekatit 12 Hospital Medical College and Menelik II comprehensive specialized hospital medical referral clinics among hypertensive patients on follow-up from November 1, 2022, to February 28, 2023.

These two hospitals were selected because they are among the largest public hospitals in Addis Ababa, offering chronic follow-up services and serving a diverse patient population. Their high patient volume and accessibility made them suitable for recruiting a representative sample.

Yekatit 12 Hospital Medical College, formerly Bethsaida Hospital established in 1915, is located in the Arada Subcity of Addis Ababa. It serves approximately 230,000 people annually in both the emergency and outpatient departments. Menelik II Comprehensive Specialized Hospital was established in 1884 by the Russian Red Cross Society as a nursing school. Progressively, it was named as Menelik II referral hospital and started working under the city government of the Addis Ababa Health Bureau.

### 2.2. Study Participants

All hypertensive patients on follow-up at Yekatit 12 Hospital Medical College and Menelik II Comprehensive Specialized Hospital medical referral clinics were used as the source population, and patients receiving antihypertensive treatment were the study population. All patients ≥ 18 years of age with hypertension and on antihypertensive treatment for at least 6 months were included in the study. The exclusion criteria were refusal to provide informed consent, pregnancy, and known mental illness.

### 2.3. Data Collection Tools and Procedures

A structured questionnaire and checklist were developed after a review of relevant works in the literature. Several questions that can address the objective of this study were constructed, including independent variables such as sociodemographic characteristics (age, sex, educational status, occupational status, income, marital status, ethnicity, religion, and residence), behavioral characteristics (medication adherence, low salt diet adherence, physical activity status, alcohol status, and smoking status), clinical characteristics (duration of hypertension, family history of hypertension, availability of BP cuff at home, BP monitoring at home or elsewhere, comorbidity, and number of antihypertensive drugs), and dependent variables such as antihypertensive drug adherence and blood pressure control. The tool was amended after a pretest was performed on 5% of patients having follow-up for hypertension at St. Paul's Hospital Millennium Medical College. The data were collected by general practitioners working in the chronic medical follow-up clinic of the hospital from November 1, 2022, to February 28, 2023.

### 2.4. Sample Size and Statistical Analysis

The sample size was calculated using Cochran's formula, assuming an overall good medication adherence rate of 51.9% according to the most comparable recent study conducted in Dessie Referral Hospital [10], Ethiopia, which is a public healthcare facility serving patients with similar demographics to our current study sites. A 95% confidence interval (CI) and a 5% margin of error (d) were used to calculate the sample size.

(1) $$\begin{array}{c} n=\frac{Z_{{\alpha /2}^{2}}\left(p\right)\left(1-p\right)}{d^{2}}=\frac{1.96^{2}\times 0.519\times \left(1-0.519\right)}{{\left(0.05\right)}^{2}}=384. \end{array}$$

Since the total population was 4420, a correction formula was used, and a 10% nonresponse rate was assumed. The final sample size was calculated to be 393. Study participants were selected using systematic random sampling. After the first patient was selected randomly, every 13th patient who visited the follow-up clinic was selected until the required sample size was obtained.

After checking the data for consistency, completeness, clarity, and accuracy, they were coded manually, entered into Epi-Info version 7.2.1, and exported to Statistical Package for Social Sciences (SPSS) Version 25.0 for analysis. After descriptive statistics, bivariate analysis was performed to determine the associations between the independent and dependent variables. Variables with a *p*-value less than 0.25 in the bivariate analysis were included in the multivariate logistic regression model to identify independent predictors. Prior to conducting the multivariate analysis, multicollinearity among the independent variables was assessed using the variance inflation factor (VIF), and all VIF values were below 2, indicating no significant multicollinearity. Backward stepwise regression was chosen to identify the most parsimonious model while minimizing the risk of overfitting, especially given the number of candidate variables. This method systematically removes nonsignificant variables to retain only those that contribute meaningfully to the model. A *p*-value of less than 0.05 was considered statistically significant, and adjusted odds ratios (AORs) with 95% CIs were reported to describe the strength and direction of associations.

### 2.5. Operational Definitions

#### 2.5.1. Hypertension

Hypertension was defined as an office SBP ≥ 140 mmHg and/or a DBP ≥ 90 mmHg [1].

#### 2.5.2. Controlled Blood Pressure

Controlled blood pressure was defined as SBP < 130 and DBP < 80 in accordance with the 2023 European Society of Hypertension (ESH) Guidelines, which recommend this threshold as the target for most hypertensive patients, particularly those at elevated cardiovascular risk [1].

#### 2.5.3. Drug Adherence

Drug adherence was defined based on Hill-Bone Medication Adherence Scale (HB-MAS) [20], which has been validated in Ethiopian hypertensive patients with Cronbach's alpha of 0.806 and predictive validity correlating modestly with SBP, supporting its reliability and cultural appropriateness [21]:

• Patients were considered adherent to their medication(s) when HB-MAS score was > 18.
• Nonadherence was considered when the patient's HB-MAS score was ≤ 18.

### 2.6. Ethics Approval and Consent to Participate

Ethical approval was obtained from the Ethics Review Committee of the Addis Ababa City Administration Health Bureau (Ref No: 19/19/3148/227) and the Ethics Committee of Yekatit 12 Hospital Medical College (Ref No: 381/22). Written informed consent was obtained from the study participants. Participant identity remains confidential. The answers received were documented and analyzed anonymously.

## 3. Results

### 3.1. Sociodemographic Characteristics

A total of 393 participants were included in the study with a 100% response rate. As detailed in Table 1, the majority of the respondents were between 46 and 60 years (42%), and the mean age of the patients was 59.2 (±12.1 SD) years. More than half (57.3%) of the study participants were female. Approximately 25% of the respondents completed college/university. Those who lived in urban areas constituted the majority of participants (98%), and approximately 45.8% of them had a monthly total income greater than 3000 Ethiopian birr (ETB) per month, followed by those who earned 1000–2000 ETB (22.4%) (Table 1).

### 3.2. Behavioral Characteristics

Of the 393 participants, 4.6% were active cigarette smokers. The prevalence of alcohol consumption was 22.9%. Most of the respondents ate vegetables (44%) and cereal products (42.7%) over the past 6 months on most days of the week. More than half (59.5%) of the respondents used additional salt in their diet. A quarter (75.1%) of the respondents had moderate physical activity, with a median duration of 30 min per day (Table 2).

### 3.3. Comorbidities

Considering the duration of hypertension, in almost half (49.6%) of the patients, hypertension was diagnosed within the past 5 years. Diabetes mellitus (49.1%) and heart failure (19.8%) were the two most common comorbidities. More than one-third of the respondents had a family history of hypertension (Table 3).

### 3.4. Antihypertensive Drug Utilization

CCBs (67.2%), ACEIs (58.8%), and thiazide diuretics (26.5%) are the three most commonly utilized antihypertensive drugs. ACEIs and CCBSs are the most common combination treatment, accounting for approximately 33.8%. Monotherapy was utilized in 37.2% of patients, whereas polytherapy was utilized in 62.8% of study participants. Adverse drug reactions were reported in five (1.3%) patients; two of these reactions were due to CCBs and ACEIs, and for one patient, no specific drug was identified. Most participants (95.4%) bought antihypertensive medication from hospitals and received physician advice about the benefits of antihypertensive drugs (Table 4).

The respondent's level of adherence to antihypertensive drugs was assessed using the eight-item HB-MAS. It was found that 72.5% of the respondents were adherent to medication, whereas 27.5% were not.

Blood pressure was measured at home in more than half (52.4%) of the patients. The rates of blood pressure control before 6 months, after 3 months, and at the time of the study were 20.4%, 27.2%, and 23.4%, respectively, and the rates of recent blood pressure control at Yekatit 12 Hospital Medical College and Menelik II Referral Hospital were 28.8% and 11.5%, respectively.

### 3.5. Factors Associated With Antihypertensive Drug Adherence

A multiple logistic regression model was used to assess the associations between adherence status and various factors. All statistically significant variables from univariate analysis at a level of 0.25 were included in the multiple logistic regression analysis to control for confounding effects. The 13 factors that were included in the model were sex, age, level of education, monthly income, number of antihypertensive medications, additional salt on a plate, vigorous physical exercise, moderate physical exercise, duration of hypertension, CVDs, chronic kidney disease, blood pressure measurement at home, and blood pressure control status.

According to the above table, participants who consumed additional salt on the plate were 3.3 times more likely to adhere than those who did not consume additional salt on the plate (odds ratio [OR], 3.31; 95% CI, 1.84–5.96), participants who consumed single antihypertensive medications were 47% less likely to adhere than those who consumed two or more antihypertensive medications (odds ratio [OR], 0.53; 95% CI, 0.30–0.94), participants who did not perform moderate physical exercise were 70% less likely to adhere than those who were engaged in moderate physical exercise (odds ratio [OR], 0.3; 95% CI, 0.20–0.65), and participants without chronic kidney disease were 3.8 times more likely to be nonadherent than those with chronic kidney disease (odds ratio [OR], 3.85; 95% CI, 1.41–10.52).

According to the bivariate regression analysis, 41.7% of the patients with uncontrolled blood pressure were adherent to their blood pressure less than those with controlled blood pressure (*p* = 0.041, *p* < 0.05) (odds ratio [OR], 0.59; 95% CI, 0.36–0.97) (Table 5).

### 3.6. Factors Associated With Blood Pressure Control

The associations between blood pressure control and patient-related, medication-related, and institution-related factors were assessed using bivariate and multivariate logistic regression. Multiple logistic regression models were used to assess the adjusted associations of predictors with adherence status. All statistically significant variables from bivariate analysis at a level of 0.25 were included in the multiple logistic regression analysis to control for confounding effects. The 10 factors that were included in the model were sex, cigarette smoking status, alcohol consumption status, diet, type of oil used, duration of hypertension, incidence of CVD, health education about hypertension and its treatment, drug adherence, and blood pressure measurement at home. Alcohol consumption, male sex, and blood pressure measurement at home were found to be independent predictors of controlled blood pressure.

As shown in Table 6, male participants had 1.95 times more controlled blood pressure than female participants (odds ratio [AOR], 1.95; 95% CI, 1.04–3.28), participants who did not drink alcohol had 1.92 times more controlled blood pressure than those who drink alcohol (AOR, 1.92; 95% CI, 1.05–3.49), and participants who did not measure blood pressure at home had 41% less controlled blood pressure than those who measure blood pressure at home (odds ratio [AOR], 0.59; 95% CI, 0.36–0.99) (Table 6).

## 4. Discussion

Despite the presence of effective interventions to lower blood pressure, hypertension control remains unacceptably low in LMICs. This study assessed the medication adherence rate and blood pressure control among hypertensive patients in Ethiopia. The majority of the study participants were adherent to the antihypertensive medications. However, despite this good adherence, less than one-third of the participants had controlled blood pressure. The addition of salt on the plate, moderate physical exertion, chronic kidney disease, and the number of antihypertensive drugs were strongly associated with adherence, whereas male sex, alcohol consumption, and blood pressure measurement at home were significantly associated with good blood pressure control. Antihypertensive drug adherence was significantly associated with controlled blood pressure.

In this study, the rate of antihypertensive drug adherence was 72.5%, which is higher than that reported in other studies conducted in Ethiopia including Dessie Referral Hospital (51.9%) [22], Jimma University Medical Center (61.8%) [23], Southwest Ethiopia (60.5%) [24], Nedjo General Hospital (31.4%) [25], and the national medication adherence rate among hypertensive patients (65.1%) [26]. However, the rate of medication adherence was comparable to that in a study conducted in Sudan, which was 70.5% [27], and lower than that in a study from Tanzania, where the adherence rate was 76.9%, and a study in Japan reported an adherence rate of 91.5% [28].

The variation from other countries could be due to socioeconomic factors and population characteristics, and from previous studies in our country, it could be that most of the studies were performed in rural areas where most of the residents did not attend formal education. This study was conducted in an urban setting where society is perceived to have a better awareness of drug adherence. The other reason could be the difference in the assessment tools used.

The rate of blood pressure control was 23.4%, which is lower than that reported in studies conducted in Southwest Ethiopia (50.3%) [24], Jimma University Medical Center (42.8%) [29], the University of Gondar Referral Hospital (50.4%) [30], Dessie city (55.8%) [31], Eastern Sudan (45.3%) [27], and Cameroon (36.8%) [32]. However, this study has results comparable to those of the US study (24%) [33], which used a similar operational definition of controlled hypertension.

The variation might be due to the difference in the operational definition of controlled blood pressure in previous studies, which was SBP less than 140 and a DBP less than 90, compared to the operational definition in this study, which was SBP less than 130 and a DBP less than 80, aligning with the 2023 ESH guideline.

The low rate of blood pressure control, despite good medication adherence, may reflect broader health system challenges. Inconsistent availability of antihypertensive medications, limited access to routine follow-up care, inadequate counseling on lifestyle changes, and financial barriers to purchasing medications or home BP monitors may all contribute to poor BP control. These systemic issues highlight the need for a more integrated and patient-centered approach to hypertension management in resource-limited settings.

A statistically significant association was not found between adherence to antihypertensive medication and patient-related factors, including age, sex, level of education, marital status, residence, income, and occupation, which is similar to the findings of studies conducted at Jimma University Medical Center [29], Eastern Sudan [27], and Cameroon [32].

A statistically significant association was found between the adherence to antihypertensive medication and the addition of additional salt to the plate. Those participants who added additional salt to the plate were more adherent (*p* < 0.01). This may reflect behavioral prioritization, in which patients focus more on medication intake than on challenging lifestyle changes such as dietary modification. Alternatively, social desirability bias may have led to underreporting of dietary adherence. In this study, the rate of dietary salt restriction was 40%, which is higher than that reported in two meta-analysis and systematic review studies, which was 34% [34] and 37.3% [35]. However, no data have shown an association with medication adherence.

Participants who took single antihypertensive medications were less adherent than those who took two or more antihypertensive medications. Similar findings were reported in Nigeria, in which patients taking three or more antihypertensive medications were more adherent at a rate of 73.1% [36], and a study from Lebanon demonstrated that taking two or more drugs was a predictor of high medication adherence (*p* = 0.02) [37]. Another study from Pakistan also reported that patients on monotherapy had a mean adherence of 79%, compared to 90% for those on three drugs or more antihypertensive medications [38]. However, a study conducted at Jimma University Medical Center showed that the use of two or more antihypertensive medications was associated with poor medication adherence [23], and a cross-sectional study conducted in Saudi Arabia showed that the use of multiple medications was associated with medication nonadherence (*p* < 0.01) [39].

CKD was associated with good medication adherence in our study. The opposite results were reported in various studies in which the presence of chronic kidney disease was associated with poor drug adherence, and the adherence rate also decreased with worsening CKD [40–42]. Although polypharmacy in CKD patients is often linked to poor adherence, our study found that participants with CKD were significantly more likely to adhere to antihypertensive medications. This may reflect an increased health system contact, closer follow-up, and heightened patient awareness of disease severity. CKD patients in tertiary settings often receive more frequent education and monitoring, which can positively influence adherence. Similar findings have been reported in settings where comorbid conditions are associated with better adherence due to increased perceived risk and care engagement. Notably, a Brazilian study showed that drug adherence tends to improve as renal function deteriorates [43], and another systematic review and meta-analysis revealed a 2.54-fold increase in the odds of being adherent among patients who had comorbidities compared with those without comorbidities [26].

In this study, male respondents had a greater rate of blood pressure control than females did. However, an opposite finding was reported in a study conducted in Kenya, in which male sex was found to be an independent predictor of poor blood pressure control [44]. A study from the US also reported that male sex was independently associated with uncontrolled blood pressure [45].

Participants who did not drink alcohol had good blood pressure control in this study. REasons for Geographic and Racial Differences in Stroke (REGARDS) Study also showed that heavy alcohol consumption is associated with poor BP control (*p* = 0.02) [42]. Population-based studies conducted in the UK showed that higher alcohol use was associated with poor blood pressure control [46].

Participants who measured blood pressure at home had more controlled blood pressure. Similar findings were reported from a systematic review and meta-analysis by Cappuccio FP; blood pressure was lower in people with hypertension who had home blood pressure monitoring (HBPM) than in those who had standard blood pressure monitoring in the healthcare system [47].

Blood pressure control is found to be associated with medication adherence. Similar findings were reported in several studies demonstrating the strong association between antihypertensive drug adherence and blood pressure control [26, 48–50].

This study has several limitations that should be acknowledged. First, the use of indirect methods to assess medication adherence, specifically self-reporting via the Hill-Bone scale, may be subject to recall and social desirability biases. Similarly, HBPM data were based on patient self-report, and we did not validate home measurements against standard clinic-based methods. The type, accuracy, and calibration status of home BP devices were also not assessed, potentially introducing variability in BP readings. Furthermore, as a cross-sectional observational study, our findings are limited to associations and do not allow for a causal inference between the identified predictors and outcomes such as blood pressure control or medication adherence. Finally, the possibility of residual confounding from unmeasured variables cannot be excluded.

## 5. Conclusion

Antihypertensive drug adherence is the most important management component for hypertensive patients to achieve adequate blood pressure control, which is important in reducing hypertension-associated adverse cardiovascular morbidity and mortality. It was found that 72.4% of patients were adherent to antihypertensive medications although only 23.4% of patients had controlled blood pressure. This signifies that in addition to pharmacologic treatment, lifestyle modification plays a critical role in achieving optimal blood pressure control. Therefore, public health policies should prioritize strengthening primary care systems to provide integrated hypertension management, including lifestyle counseling, dietary support, and affordable access to medications and monitoring tools.

## Figures and Tables

**Table 1:** Sociodemographic characteristics of the patients (*n* = 393).

Characteristics	Frequency (*n*)	Percentage (%)
Sex		
Male	168	42.7
Female	225	57.3
Age in years		
18–44	48	12.2
45–60	165	42.0
61–75	146	37.2
> 76	34	8.7
Educational level		
Unable to read and write	65	16.5
Read and write	26	6.6
Primary school	67	17.0
Secondary school	68	17.3
High school/preparatory	68	17.3
College/university	99	25.2
Marital status		
Single	30	7.6
Married	253	64.4
Divorced	25	6.4
Widowed	85	21.6
Occupation		
Government employee	58	14.8
Retired	88	22.4
Housewife	122	31.0
Private	85	21.6
No job	40	10.2
Residence		
Urban	385	98
Rural	8	2
Monthly income in ETB		
< 1000	52	13.2
1000–2000	88	22.4
2001–3000	73	18.6
> 3000	180	45.8

**Table 2:** Behavioral characteristics of the patients (*n* = 393).

Behavioral character	Frequency (*n*)	Percentage (%)
Cigarette smoking		
Yes	18	4.6
No	375	95.4
Alcohol consumption		
Yes	90	22.9
No	303	77.1
Additional salt added on plate		
Yes	234	59.5
No	159	40.5
Vigorous physical exercise		
Yes	17	4.3
No	376	95.7
Moderate physical exercise		
Yes	295	75.1
No	98	24.9

**Table 3:** Duration of hypertension and comorbidities of the patients (*n* = 393).

	Frequency (*n*)	Percentage (%)
Duration of hypertension		
< 5 years	195	49.6
5–10 years	120	30.5
> 10 years	78	19.8
Comorbidities		
Cardiovascular diseases	78	19.8
Diabetes mellitus	193	49.1
Chronic kidney disease	23	5.9
Asthma	14	3.6
Family history of hypertension		
Yes	149	37.9
No	244	62.1

**Table 4:** Antihypertensive drug utilization of the patients (*n* = 393).

Antihypertensive drugs	Frequency (*n*)	Percentage (%)
CCBs	264	67.2
ACEIs	231	58.8
Thiazide diuretics	104	26.5
Beta blockers	56	14.2
Loop diuretics	49	12.5
ARBs	8	2
MRAs	18	4.6
ACEIs + CCBs	133	33.8
ACEIs + thiazide diuretics	47	12
CCBs + thiazide diuretics	55	14
ACEIs + CCBs + thiazide diuretics	23	5.9
Monotherapy	146	37.2
Polytherapy	247	62.8
Drug availability in hospital		
Yes	375	95.4
No	18	4.6
Physician advice about the benefits of antihypertensive drugs		
Yes	334	85.0
No	59	15.0
Average cost of antihypertensive drugs	600.53 birr	

**Table 5:** Factors associated with antihypertensive drug adherence.

Variable	Drug adherence		COR (95% CI)	*p* value	AOR (95% CI)	*p* value
	Yes	No				
Sex						
Male	127	41	0.76 (0.48–1.19)	0.23	0.74 (0.376–1.45)	0.38
Female	158	67	1			
Age						
25–44	29	19	1.2 (0.48–2.9)	0.69		
45–60	124	41	0.6 (0.27–1.3)	0.21	1.04 (0.37–2.94)	0.92
61–75	110	36	0.6 (0.27–1.3)	0.20	1.44 (0.55–3.77)	0.45
76–90	22	12	1			
Level of education						
Unable to read and write	44	21	2.14 (1.03–4.45)	**0.04**	1.02 (0.35–2.95)	0.95
Read and write	15	11	3.30 (1.30–8.37)	**0.01**	0.68 (0.20–2.28)	0.53
Primary school	51	16	1.41 (0.66–3.01)	0.37		
Secondary school	47	21	2.01 (0.97–4.15)	0.05	0.67 (0.276–1.63)	0.38
High school/preparatory	47	21	2.01 (0.97–4.15)	0.05	0.42 (0.18–1.00)	0.05
College/university	81	18	1			
Occupation						
Government employee	43	15	0.81 (0.33–1.99)	0.65		
Retired	71	17	0.55 (0.23–1.31)	0.18	1.76 (0.60–5.16)	0.30
Housewife	79	43	1.27 (0.58–2.74)	0.54		
Private	64	21	0.76 (0.33–1.76)	0.53		
No job	28	12	1			
Monthly income						
< 1000	32	20	2.05 (1.06–3.96)	**0.03**	0.55 (0.23–1.29)	0.17
1000–2000	59	29	1.61 (0.92–2.83)	0.09	0.63 (0.31–1.30)	0.21
2001–3000	56	17	0.99 (0.52–1.89)	0.99		
> 3000	138	42	1			
Added salt to plate						
Yes	146	88	0.23 (0.13–0.4)	**≤ 0.001** ^**∗**^	3.31 (1.84–5.966)	**≤ 0.001** ^**∗∗**^
No	139	20				
Vigorous physical exercise						
Yes	9	8	0.40 (0.15–1.08)	0.07	2.31 (0.66–8.01)	0.18
No	276	100	1			
Moderate physical exercise						
Yes	230	65	0.76 (1.70–4.49)	**≤ 0.001** ^**∗**^	0.30 (0.20–0.65)	**0.01** ^**∗∗**^
No	55	43	1			
Duration of hypertension						
< 5 years	135	60	1.06 (0.59–1.88)	0.83		
5–10 years	95	25	0.62 (0.32–1.21)	0.16	1.17 (0.54–2.53)	0.68
> 10 years	55	23	1			
Cardiovascular disease						
Yes	49	29	0.56 (0.33–0.97)	**0.03**	1.40 (0.73–2.67)	0.30
No	236	79	1			
CKD						
Yes	9	14	0.21 (0.08–0.51)	**≤ 0.001** ^**∗**^	3.85 (1.41–10.52)	**0.01** ^**∗∗**^
No	276	94	1			
Health education						
Yes	44	23	1.48 (0.84–2.59)	0.17	0.82 (0.42–1.59)	0.56
No	241	85	1			
Number of antihypertensive drugs						
Single	118	28	0.49 (0.30–0.80)	**≤ 0.001** ^**∗**^	0.53 (0.30–0.94)	**0.03** ^**∗∗**^
Two and above	167	80	1			
BP measurement at home						
Yes	161	45	1.81 (1.16–2.84)	**≤ 0.001**	0.84 (0.48–1.46)	0.54
No	124	63				
Blood pressure control						
Controlled	59	33	0.59 (0.36–0.97)	**0.04**	1.58 (0.87–2.85)	0.12
Not controlled	226	75				

Note: Bold values signify statistical significance. Statistically significant values in both the AOR and COR are marked by (^∗^) in the AOR and by (^∗∗^) in the COR.

Abbreviations: AOR = adjusted odds ratio, CI = confidence interval, COR = crude odds ratio.

**Table 6:** Factors associated with blood pressure control.

Variable	BP control		COR (95% CI)	*p*-value	AOR (95% CI)	*p*-value
	Yes	No				
Sex						
Male	27	141	0.3 (0.28–0.71)	**0.004** ^**∗**^	1.854 (1.04–3.28)	**0.035** ^**∗∗**^
Female	65	160	1			
Cigarette smoking						
Yes	91	284	6.66 (0.75–58.8)	0.08	0.15 (0.01–1.28)	0.08
No	1	17	1			
Alcohol drinking						
Yes	63	240	0.44 (0.23–0.83)	**0.01** ^**∗**^	1.92 (1.05–3.49)	**0.031** ^**∗∗**^
No	29	61	1			
Diet						
Meat	12	40	1.16 (0.47–2.90)	0.73		
Vegetables	48	125	1.75 (0.96–3.21)	0.06	0.69 (0.40–1.21)	0.20
Cereal products	32	136	1			
Type of oil						
Vegetable oil	87	276	3.22 (0.55–18.67)	0.19	0.41 (0.08–1.94)	0.26
Butter	1	4	2.74 (0.14–50.73)	0.49		
Sesame/nug oil	2	5	2.30 (0.17–30.57)	0.52		
Others	2	16	1			
Duration of hypertension						
< 5 years	49	146	0.66 (0.32–1.37)	0.27		
5–10 years	21	99	0.49 (0.22–1.09)	0.08	1.90 (0.91–3.96)	0.08
> 10 years	22	56	1			
Cardiovascular disease						
Yes	68	247	0.52 (0.26–1.00)	0.05	1.77 (0.96–3.24)	0.06
No	24	54				
Health education						
Yes	21	46	1.68 (0.87–3.25)	0.12	0.67 (0.36–1.25)	0.21
No	71	255	1			
Drug adherence			.			
Yes	59	226	0.56 (0.30–1.03)	0.06	1.4 (0.48–1.46)	0.21
No	33	75	1			
BP measurement at home						
Yes	56	131	1.86 (1.05–3.30)	**0.03** ^**∗**^	0.59 (0.36–0.99)	**0.04** ^**∗∗**^
No	36	170	1			

Note: Statistically significant values in both the AOR and COR are marked by (^∗^) in the AOR and by (^∗∗^) in the COR.

Abbreviations: AOR = adjusted odds ratio, CI = confidence interval, COR = crude odds ratio.

## Data Availability

The data that support the findings of this study are available from the corresponding author upon reasonable request.
